# Hydrogen peroxide suppresses excitability of gonadotropin-releasing hormone neurons in adult mouse

**DOI:** 10.3389/fendo.2022.939699

**Published:** 2022-10-28

**Authors:** Santosh Rijal, Seon Hui Jang, Dong Hyu Cho, Seong Kyu Han

**Affiliations:** ^1^ Department of Oral Physiology, School of Dentistry and Institute of Oral Bioscience, Jeonbuk National University, Jeonju, South Korea; ^2^ Department of Obstetrics and Gynecology, Jeonbuk National University Medical School, Institute of Clinical Medicine of Jeonbuk National University-Biomedical Research Institute for Medical Sciences, Jeonbuk National University Hospital, Jeonju, South Korea

**Keywords:** hydrogen peroxide, gonadotropin-releasing hormone neurons, hypothalamic-pituitary-gonadal axis, patch-clamp, K_ATP_ channels, reactive oxygen species

## Abstract

It has been reported that reactive oxygen species (ROS) derived from oxygen molecule reduction can interfere with the cross-talk between the hypothalamic-pituitary-gonadal (HPG) axis and other endocrine axes, thus affecting fertility. Furthermore, ROS have been linked to GnRH receptor signaling in gonadotropes involved in gonadotropin release. There has been evidence that ROS can interfere with the HPG axis and gonadotropin release at various levels. However, the direct effect of ROS on gonadotropin-releasing hormone (GnRH) neuron remains unclear. Thus, the objective of this study was to determine the effect of hydrogen peroxide (H_2_O_2_), an ROS source, on GnRH neuronal excitabilities in transgenic GnRH-green fluorescent protein-tagged mice using the whole-cell patch-clamp electrophysiology. In adults, H_2_O_2_ at high concentrations (mM level) hyperpolarized most GnRH neurons tested, whereas low concentrations (pM to μM) caused slight depolarization. In immature GnRH neurons, H_2_O_2_ exposure induced excitation. The sensitivity of GnRH neurons to H_2_O_2_ was increased with postnatal development. The effect of H_2_O_2_ on adult female GnRH neurons was found to be estrous cycle-dependent. Hyperpolarization mediated by H_2_O_2_ persisted in the presence of tetrodotoxin, a voltage-gated Na^+^ channel blocker, and amino-acids receptor blocking cocktail containing blockers for the ionotropic glutamate receptors, glycine receptors, and GABA_A_ receptors, indicating that H_2_O_2_ could act on GnRH neurons directly. Furthermore, glibenclamide, an ATP-sensitive K^+^ (K_ATP_) channel blocker, completely blocked H_2_O_2_-mediated hyperpolarization. Increasing endogenous H_2_O_2_ by inhibiting glutathione peroxidase decreased spontaneous activities of most GnRH neurons. We conclude that ROS can act as signaling molecules for regulating GnRH neuron’s excitability and that adult GnRH neurons are sensitive to increased ROS concentration. Results of this study demonstrate that ROS have direct modulatory effects on the HPG axis at the hypothalamic level to regulate GnRH neuron’s excitabilities.

## Introduction

Reactive oxygen species (ROS) are chemically reactive molecules or free radicals formed when oxygen molecules are reduced. Mitochondria are primary cellular organelles responsible for the production of a large amount of ROS in cells (1, 2). External sources including pollution, radiation, physical stress, alcohol abuse, cigarette smoking and vaping, drug abuse, obesity, malnutrition, lifestyle modification, and endocrine-disrupting chemicals can intensify ROS production in cells (3, 4). At the cellular level, ROS at low concentrations operate as signaling molecules (5). However, excessive levels of ROS cause oxidative stress and cell death (6). Numerous enzymatic and non-enzymatic antioxidant systems can counteract increasing concentration of ROS in cells. Enzymes such as glutathione peroxidase (GPx), superoxide dismutase, and catalase (CAT) play an enzymatic role in the degradation of ROS, while scavengers such as vitamin C, vitamin E, glutathione, carotenoids, and ubiquinone play a non-enzymatic role in the detoxification of free radicals (7, 8).

Gonadotropin-releasing hormone (GnRH) neurons are key regulators of the hypothalamic-pituitary-gonadal (HPG) axis. They play a pivotal role in the regulation of fertility *via* release of gonadotropins in mammals (9). It has been shown that ROS produced by endogenous and exogenous sources can impair reproductive function, decrease gonadal hormones, and interfere with cross-talk between the HPG axis and other endocrine axes, eventually affecting fertility (3). Furthermore, ROS are connected to GnRH receptor signaling involved in gonadotropin release of gonadotropes (10). In contrast, endogenous gonadal hormones strongly influence ROS generation in brain mitochondria (11). An external source of ROS has now emerged as a leading cause of reproductive issues such as infertility and pregnancy complications (3, 12, 13).

ROS in the brain can act as potent signaling molecules at physiological concentration. Neurons can sense, convert, and transmit ROS into relevant intracellular signals and regulate peripheral tissue activities *via* the autonomous nervous system (14). New evidence has suggested that ROS play a signaling role in regulating hypothalamus activity. For example, ROS in the hypothalamus can regulate energy homeostasis (15) and maintain body fluid dynamics (16). ROS can also affect functions of hypothalamic neurons such as neuropeptide-Y (NPY)/agouti-related protein (AgRP) neurons, pro-opiomelanocortin (POMC)/cocaine-and-amphetamine responsive transcript (CART) neurons, and paraventricular nucleus (PVN) (17, 18). Hormones, peptides, neurotransmitters, and nutrients can also affect the release of ROS in the hypothalamus (14).

Studies mentioned above have shown that ROS can inhibit gonadotropin release at several levels of the HPG axis. However, the mechanism underlying how ROS impact GnRH neuronal activities remains unknown. Among various ROS, hydrogen peroxide (H_2_O_2_) is the most stable and long-lived ROS as it has a cellular half-life of 1 ms compared to other ROS such as superoxide anion radicals (1 μs), and hydroxyl radicals (1 ns) (19–21). Furthermore, Ledo et al. reported that the extracellular H_2_O_2_ in brain slices and *in vivo* has a half-life of 2.5 and 2.2 s respectively (22). Additionally, H_2_O_2_ is a highly diffusible and less toxic ROS that has emerged as a neuromodulator and an intercellular signaling molecule in the brain (19, 22). H_2_O_2_ perfusion on brain slices can influence neuronal excitabilities (18, 23–25), synaptic activity, and neurotransmitter release (26, 27). Thus, the objective of this study was to investigate the effect of membrane diffusible extracellular ROS source H_2_O_2_ on excitabilities of GnRH neurons in hypothalamic preoptic area (hPOA) brain slices using a whole-cell patch-clamp approach.

## Materials and methods

### Animals

C57BL/6 GnRH-green fluorescent protein-tagged (GnRH-GFP) mice (28) housed under stable room temperature (23-26 °C) and an automatic 12:12-h light-dark cycle (lights on at 07:00 h) with *ad libitum* access to food and water were sacrificed for the experiment. All animal care conditions and experimental procedures were in accordance with the Institutional Animal Care and Use Committee of Jeonbuk National University (CBNU 2020-0122). Estrous cycle stage of female mice was assessed by vaginal smear examination.

### Preparation of brain slices

Coronal brain slices were prepared using the same procedure as described in a previous study (29). In brief, mice were beheaded between 10:00 and 12:00 p.m. UTC+09:00 (Universal Time Coordinated). Their brains were swiftly removed and immersed in ice-cold artificial cerebrospinal fluid (ACSF) containing 126 mM NaCl, 2.5 mM KCl, 2.4 mM CaCl_2_, 1.2 mM MgCl_2_, 11 mM D-glucose, 1.4 mM NaH_2_PO_4_, and 25 mM NaHCO_3_ (pH value of 7.3 to 7.4 was maintained when bubbled with 95% O_2_ and 5% CO_2_). Coronal brain slices (180-270 μm thick) containing the preoptic hypothalamic area were prepared using a vibratome (VT1200S, Leica biosystem, Wetzlar, Germany) in ice-cold ACSF. For recovery, the brain slices were stored in oxygenated ACSF at room temperature for at least 1hour before being transferred to the recording chamber.

### Electrophysiology

Before electrophysiological recording, brain slices were transferred to the recording chamber mounted on an upright microscope (BX51W1; Olympus, Tokyo, Japan). They were, entirely submerged, and continuously perfused (4~5 mL/min) with oxygenated ACSF. The view of the coronal slice was displayed on a video monitor. The hPOA region was identified under X10 objective magnification. Fluorescent GnRH neurons were identified under X40 objective magnification *via* brief fluorescence illumination. Identified GnRH neurons were patched under Nomarski differential interference contrast optics. Thin-wall borosilicate glass capillaries (PG52151-4, WPI, Sarasota, FL, USA) were pulled on a Flaming/Brown puller (P-97; Sutter Instruments Co., Novate, CA USA) to fabricate patch pipette. These pipettes typically displayed a tip resistance of 4 to 6 MΩ when filled with pipette solution filtered through a disposable 0.22-µM filter. The loaded pipette solution was composed of 140 mM KCl, 1mM CaCl_2_, 1 mM MgCl_2_, 10 mM HEPES, 10 mM EGTA, and 4 mM Mg-ATP (pH 7.3 with KOH). Pipette offset was set to zero before a high-resistance seal (“gigaseal”) was achieved. After a giga seal was achieved between the pipette and the neuronal membrane, negative pressure by a short suction pulse was applied to make the whole cell.

Whole-cell recorded signals were amplified with an Axopatch 200B (Molecular Devices, San Jose, CA, USA) and filtered at 1 kHz with a Bessel filter before digitizing at a rate of 1 kHz. Membrane potential changes were sampled using a Digidata 1440A interface (Molecular Devices, San Jose, CA, USA). Signals were recorded and analyzed using an Axon pClamp 10.6 data acquisition program (Molecular Devices, San Jose, CA, USA). Neurons that showed changes in membrane potential of more than 2 mV after being exposed to H_2_O_2_ were considered to have responded. All recordings were made at room temperature.

### Chemicals

Chemicals including hydrogen peroxide (H_2_O_2_), picrotoxin, strychnine hydrochloride (strychnine), glibenclamide, tetraethylammonium chloride (TEA), barium chloride (BaCl2), mercaptosuccinic acid (MCS), 3-amino-1,2,4-triazole (ATZ), and ACSF compositions were purchased from Sigma-Aldrich (St. Louis, MO, USA), except for CNQX disodium salt (CNQX), DL-AP5 (AP5), and tetrodotoxin citrate (TTX) which was bought from Tocris Bioscience (Avonmouth, Bristol, UK). Stocks were diluted (usually 1,000-fold) in ACSF to desired final concentrations before bath application. H_2_O_2_ of desired concentration was freshly prepared from stock by dripping directly to ACSF immediately before bath application.

### Data and statistical analysis

For statistical analysis, Student’s t-test and one-way ANOVA post-hoc Scheffe test were used to compare means of two and more than two experimental groups, respectively. All statistical analyses were performed using Origin 8 software (OriginLab Corp, Northampton, MA, USA). All numerical values are expressed as mean ± standard error of the mean. Results with *p*-value < 0.05 are considered to be statistically significant. Traces were plotted using Origin 8 software (OriginLab Corp, Northampton, MA, USA). Action potential firings were analyzed using a Mini-Analysis software (ver. 6.0.7; Synaptosoft Inc., Decatur, GA, USA).

## Results

### Hydrogen peroxide exposure induces variegated response in GnRH neurons

**Figure 1:**
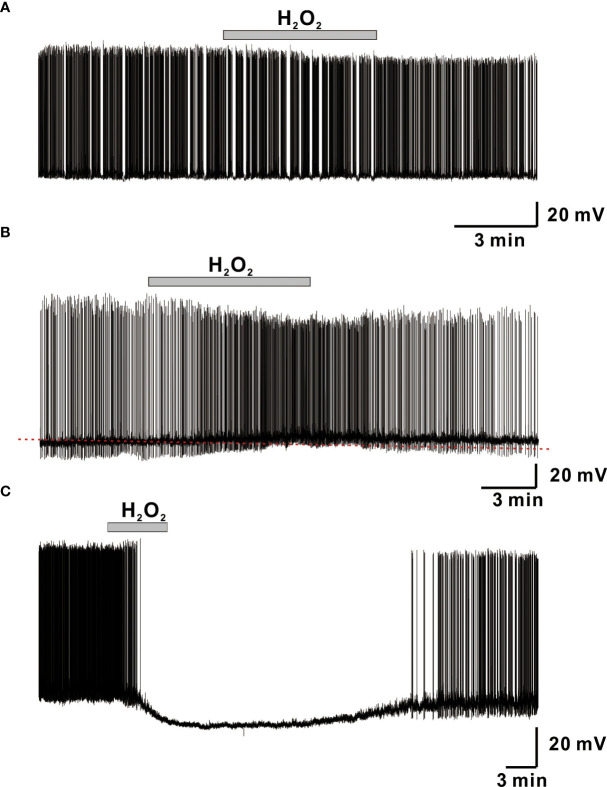
H_2_O_2_ induces variegated responses of adult male GnRH neurons. **(A–C)** Representative voltage traces from GnRH neurons showing no response, membrane depolarization, and membrane hyperpolarization, respectively, upon perfusion with 1 mM H_2_O_2_.

We used whole-cell current-clamp recordings to investigate the influence of H_2_O_2_ on membrane excitability in GnRH neurons and found that superfusion with 1 mM H_2_O_2_ elicited a variety of responses in adult GnRH neurons, including membrane hyperpolarization, depolarization, and no response as shown in **Figure 1**. Bath treatment with 1 mM H_2_O_2_ for 3 to 5 minutes produced responses in 70% of adult GnRH neurons, while 30% of adult GnRH neurons were unresponsive to H_2_O_2_ (**Figure 1A**). Among responding neurons, 10% generated an average membrane depolarization of 4.60 ± 0.65 mV (n = 15; **Figure 1B**) while 60% of neurons induced an average membrane hyperpolarization of -14.6 ± 0.81 mV (n = 82; **Figure 1C**). Depolarized neurons showed a minor increase in spontaneous action potential firing in addition to membrane potential change. In contrast, hyperpolarized neurons showed partial and/or full cessation of spontaneous action potential firing. These alterations were reversed after more than 15-20 minutes of H_2_O_2_ washout.

**Figure 2:**
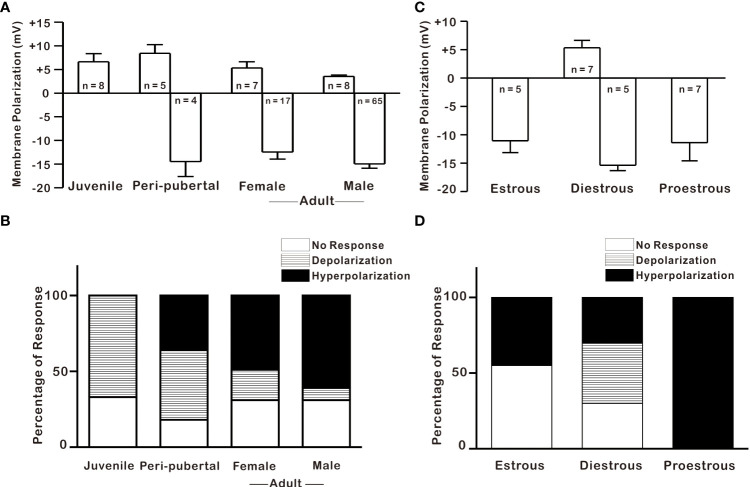
H_2_O_2_ effect on GnRH neurons across postnatal development and estrous cycle. **(A–C)** Histograms depicting H_2_O_2_-induced membrane polarization in GnRH neurons throughout the postnatal development and estrous cycle, respectively (*p* > 0.05; one-way ANOVA). **(B–D)** Histograms showing percentages of variegated responses of GnRH neurons by H_2_O_2_ exposure across postnatal development and at various estrous cycle stages in adult females, respectively.

According to previous studies, oxidative stress vulnerability increases with age, with adults being more vulnerable and juveniles being partially resistant (30, 31). In the present study, effects of 1 mM H_2_O_2_ on GnRH neurons were studied in three groups according to age: juvenile, 8 to 25 postnatal days (PND); peripubertal, 26 to 45 PND; and adults, more than 60 PND. In contrast with its hyperpolarization effect on most adult GnRH neurons, H_2_O_2_ depolarized most GnRH neurons 67% (8/12) in juveniles. On the other hand, H_2_O_2_ exposure elicited similar percentages of responses, 46% (5/11) for depolarization and 36% (4/11) for hyperpolarization in peripubertal mice as shown in **Figures 2A, B**. Furthermore, there was no significant difference in mean depolarization between juvenile and peripubertal. Similarly, GnRH neurons from both adult females and males responded equally to H_2_O_2_ exposure (females; 69%, 24/35: males; 69%, 73/106). In addition, the mean values for induced hyperpolarization (male; -14.9 ± 0.84 mV, n = 65: female; -12.5 ± 1.50 mV, n = 17, p > 0.05; unpaired *t*-test) and depolarization (male; 3.98 ± 0.46 mV, n = 8: female; 5.32 ± 1.3 mV, n = 7, p > 0.05; unpaired *t*-test) were not significantly different between adult females and males GnRH neurons as shown in **Figure 2A**. Similarly, there was no significant difference in the mean hyperpolarization among estrous phases in female mice (estrous; -11.1 ± 2.11mV, n = 5: diestrous; -15.4 ± 0.96 mV, n = 5: proestrous; -11.4 ± 3.23 mV, n = 7, p > 0.05; one-way ANOVA, **Figure 2C**). However, female GnRH neurons demonstrated estrous cycle-dependent variation in response percentage to H_2_O_2_ exposure. During H_2_O_2_ exposure, 100% of GnRH neurons from proestrous mice showed hyperpolarization, whereas only 45% of GnRH neurons from estrous mice responded to H_2_O_2_ with hyperpolarization. Similarly, 70% of GnRH neurons from diestrous mice responded to H_2_O_2_ treatment, accounting 30% for hyperpolarization and 40% for depolarization, as shown in **Figure 2D**.

### Response of adult GnRH neurons to H_2_O_2_ exposure is concentration-dependent

**Figure 3:**
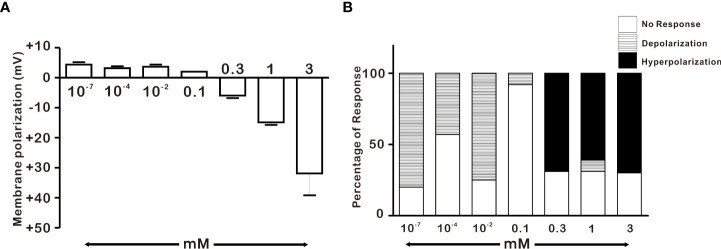
Concentration-dependent effect of H_2_O_2_ on GnRH membrane potential under whole-cell current clamp. **(A)** Histograms depicting H_2_O_2_-induced membrane polarization in response to various concentrations of H_2_O_2_ on GnRH neurons of adult males (one-way ANOVA post-hoc Scheffe test) **(B)** Histograms depicting percentage of variegated responses induced by various concentrations of H_2_O_2_ on GnRH neurons of adult males.

After discovering that adult GnRH neurons were susceptible to 1 mM H_2_O_2_, we conducted a dose-dependent experiment in adult male GnRH neurons with low and high concentrations of H_2_O_2_. As demonstrated in **Figure 3A**, low concentrations of H_2_O_2_ caused minor membrane depolarization, whereas high concentrations of H_2_O_2_ caused membrane potential to become more hyperpolarized. Low concentrations of H_2_O_2_ (100 pm, 100 nM, and 10 μM) exhibited depolarization in the majority of GnRH neurons, corresponding to 80% (8/10), 43% (3/7), and 75% (3/4), respectively. In contrast, high concentrations of H_2_O_2_ (0.3, 1 and 3 mM) induced hyperpolarization in majority of GnRH neurons, corresponding to 69% (9/13), 61% (65/106), and 72% (13/18), respectively. However, 100 μM H_2_O_2_ induced depolarization in one of the fourteen neurons tested accounting 7% as shown in **Figure 3B**.

### H_2_O_2_ acts on GnRH neurons postsynaptically

**Figure 4:**
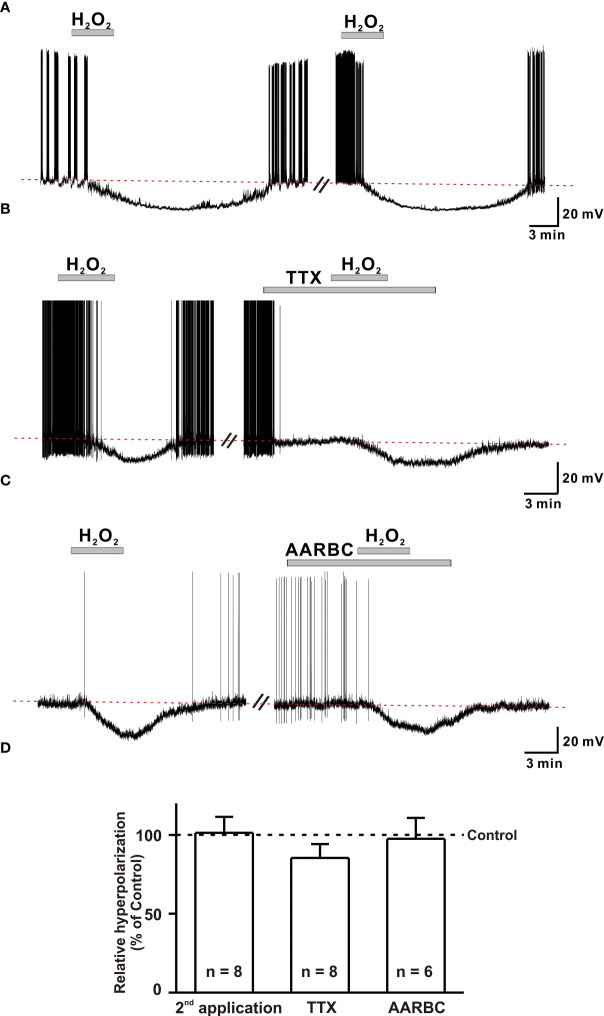
H_2_O_2_ acts on GnRH neurons post-synaptically. **(A)** A representative trace showing repeatable hyperpolarization induced by 1 mM H_2_O_2_ under a whole-cell current clamp. **(B, C)** Representative traces showing persistence of H_2_O_2_ induced hyperpolarization response in the presence of (TTX, 0.5 μM), the voltage-sensitive Na+ channel blocker and amino-acid receptor blocking cock-tail (AARBC), respectively. **(D)** A bar diagram showing mean relative values of hyperpolarization induced by 1 mM H_2_O_2_ on 2^nd^ application, in the presence of TTX, and in the presence of AARBC (*p* > 0.05; paired *t*-test).

Hyperpolarization of GnRH neurons induced by 1 mM H_2_O_2_ recovered almost completely after more than 15 to 20 minutes of washout. Therefore, we determined whether H_2_O_2_ elicited repeatable responses of GnRH neurons. To access this, H_2_O_2_ was consecutively applied after the washout of the first application. On repeat application, H_2_O_2_ induced hyperpolarization with comparable amplitude to that of the first application. The mean hyperpolarization induced by H_2_O_2_ on the first application (-18.0 ± 4.84 mV, n = 8) was similar to that induced on the second application (-18.4 ± 4.8 mV, n = 8, *p* > 0.05; **Figure 4A**). Further, we aimed to examine whether H_2_O_2_ could act on GnRH neurons directly. For this, the hyperpolarization induced on bath application of H_2_O_2_ was recorded in the presence of TTX (0.5 µM), a sodium channel blocker known to block action potential-dependent transmission. Action potentials were promptly suppressed when recorded in the presence of TTX. However, the hyperpolarizing effect of H_2_O_2_ on GnRH neurons persisted. Average responses generated by H_2_O_2_ alone (-16.8 ± 2.2 mV, n = 8) and in the presence of TTX (-13.6 ± 1.7 mV, n = 8, p > 0.05; **Figure 4B**) were not significantly different.

Next, to assess the possible involvement of both pre- and post-synaptic GABA, glycine, and glutamate receptors in H_2_O_2_ mediated actions of GnRH neurons, H_2_O_2_-induced hyperpolarization was recorded in the presence of an amino acid receptor blocker cocktail (AARBC) containing picrotoxin (50 µM), AP5 (20 µM), CNQX (10 µM), and strychnine (2 µM). Under these circumstances, H_2_O_2_ still induced hyperpolarization of GnRH neurons. The average hyperpolarization induced by H_2_O_2_ alone was -17.0 ± 1.95 mV (n = 6), which was not significantly different from that induced by H_2_O_2_ in the presence of AARBC (-16.5 ± 2.57 mV, n = 6, p > 0.05; **Figure 4C**). As shown in **Figure 4D**, the average relative percentage of H_2_O_2_-induced hyperpolarization on the second application, TTX and AARBC compared to respective control were 101.3 ± 10.1% (n = 8, *p* > 0.05), 85.3 ± 8.9% (n = 8, *p* > 0.05), and 97.5 ± 13.3% (n = 6, p > 0.05), respectively. These findings imply that H_2_O_2_ directly acts on postsynaptic GnRH neurons to induce hyperpolarization effect.

### H_2_O_2_-mediated hyperpolarization is due to activation of K_ATP_ channels

**Figure 5:**
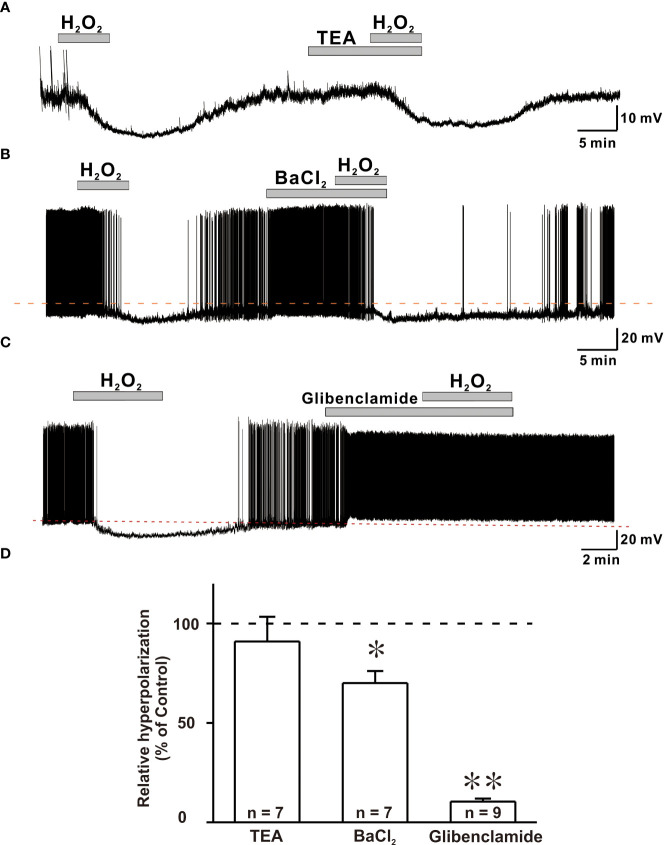
ATP-sensitive potassium channels (KATP) are susceptible to H2O2-induced hyperpolarization in GnRH neurons. **(A, B)** Representative traces showing persistence of H2O2-induced hyperpolarization response in the presence of TEA and BaCl2, respectively. **(C)** A representative trace showing complete blockade of hyperpolarization induced by 1mM H2O2 by KATP channel blocker glibenclamide under whole-cell current clamp. **(D)** A bar diagram depicting mean relative values of hyperpolarization caused by 1 mM H2O2 in the presence of various potassium channel blockers (TEA: n = 7, no significant; BaCl2: n = 7, *p < 0.05; glibenclamide: n = 9, **p < 0.01, paired t-test).

When exposed to exogenous H_2_O_2_, hyperpolarization and reduced excitation are hypothesized to be caused by the activation of potassium channels in various neuronal cells (18, 23). As a result, we examined hyperpolarization caused by H_2_O_2_ exposure in the presence of potassium channel blockers such as TEA, BaCl_2_, and glibenclamide. Blocker concentrations utilized in this study have been shown to be able to inhibit potassium channels in brain slices (32–34). To confirm the involvement of potassium channels in the hyperpolarizing effect of H_2_O_2_, the response elicited by H_2_O_2_ was examined in the presence of non-specific K^+^ channel blocker, TEA. The hyperpolarizing impact of H_2_O_2_ was maintained even in the presence of TEA (**Figure 5A**).

Next, hyperpolarization induced by H_2_O_2_ exposure was recorded in the presence of BaCl_2_, a broad-spectrum potassium channel blocker. In the presence of BaCl_2_, the hyperpolarization induced by H_2_O_2_ was partially suppressed (**Figure 5B**). Next, glibenclamide, K_ATP_ channel blocker, was coapplied with H_2_O_2_. After treatment with glibenclamide, five of nine GnRH neurons depolarized with increased firing frequency. Glibenclamide also prevented H_2_O_2_-elicited hyperpolarization of all neurons examined (**Figure 5C**). As shown in **Figure 5D**, average relative hyperpolarization percentages induced by H_2_O_2_ in the presence of TEA, BaCl_2_ and glibenclamide compared to those by H_2_O_2_ alone were 91.0 ± 12.4% (n = 7, *p* > 0.05), 70.0 ± 6.04% (n = 7, **p < 0.01), and 10.5 ± 1.52% (n = 9, ***p < 0.001), respectively. These findings imply a complete involvement of K_ATP_ channels in H_2_O_2_-mediated hyperpolarization of GnRH neurons.

### Role of endogenous H_2_O_2_ in regulating excitability of GnRH neurons

**Figure 6:**
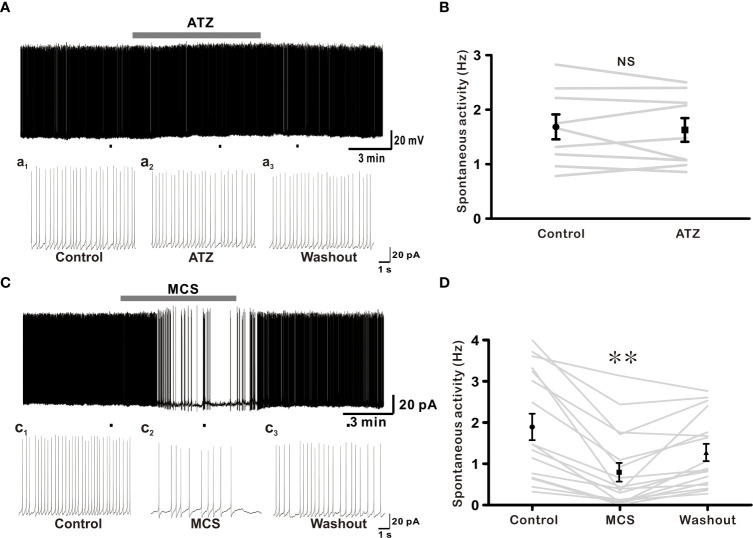
Glutathione peroxidase (GPx) inhibition suppresses excitability of GnRH neurons. **(A)** A representative current-clamp trace showing no effect of 1 mM ATZ (catalase inhibitor) on GnRH neurons. **(C)** A typical current-clamp trace showing a decrease of spontaneous activity of GnRH neurons after perfusion with 1 mM MCS, a GPx inhibitor. **(B, D)** Before and after plot showing effects of ATZ and MCS on mean spontaneous firing of GnRH neurons, respectively (**p < 0.01; one-way ANOVA).

In this study, exogenous H_2_O_2_ was identified as a possible regulator of GnRH neuron activity, influencing membrane potential and spontaneous firing activities. Next, we determined whether elevation in endogenously produced H_2_O_2_ could affect the activity of these cells. Recent studies have shown that endogenous H_2_O_2_ amplification can regulate neuronal excitability in distinct neuronal populations (23, 35).To explore the influence of endogenous H_2_O_2_ on GnRH neurons excitability, ATZ (1 mM), a CAT inhibitor, and MCS (1 mM), a GPx inhibitor, were bath applied. ATZ and MCS have been shown to increase the production of intracellular H_2_O_2_ in cells (23, 35). Using ATZ, we first examined the effect of CAT inhibition on GnRH neuronal activity. Except for one neuron that displayed depolarization of 19.7 mV, bath administration of 1mM ATZ had no significant effect on membrane potential or spontaneous activity of all GnRH neurons examined (**Figure 6A**). The frequency of spontaneous firing under ATZ treatment remained considerably unaltered compared to that of the control as shown in **Figure 6B** (Control: 1.68 ± 0.229, ATZ: 1.63 ± 0.22; n = 9; *p* > 0.05). Inhibiting GPx with MCS resulted in a partial cessation of spontaneous activity in most (13/17) GnRH neurons and a complete blockade in four neurons. In the presence of MCS, the spontaneous firing activity of GnRH neurons decreased from 1.90 ± 0.32 Hz to 0.80 ± 0.23 Hz (n = 17; *p* < 0.05; **Figures 6C, D**), with an average decrease of 66.2 ± 5.2%. In addition, MCS exposure resulted in membrane response in 9 of 17 GnRH neurons tested. Among them, seven neurons displayed a slight depolarization (3.75 ± 0.43 mV, n = 7), whereas the remaining two exhibited hyperpolarization of -3.70 ± 0.67 mV. All changes were reversible upon washout of MCS with ACSF.

## Discussion

For the first time, this study shows that the majority of adult GnRH neurons are vulnerable to oxidative stress. This study aimed to determine the role of ROS H_2_O_2_ in modulating the GnRH neuronal activity. Our electrophysiological data demonstrated that exogenous H_2_O_2_ elicited post-synaptic inhibition of activities of most adult GnRH neurons *via* activation of K_ATP_ channels. Furthermore, immature GnRH neurons, unlike adult GnRH neurons, exhibited excitation upon H_2_O_2_ exposure. The vulnerability of GnRH neurons to H_2_O_2_ increased with postnatal development. H_2_O_2_ sensitivity to adult GnRH neurons was found to be highly dependent on H_2_O_2_ concentration and the estrous cycle of females. In addition, inhibiting GPx caused GnRH neurons to lose their spontaneous activity.

The hypothalamus is a predominant brain area that receives integrated information from multiple sources, including hormones, neurotransmitters, and metabolites, to regulate homeostasis, energy metabolism, and hormone release (14, 36). Furthermore, the hypothalamus is highly susceptible to oxidative stress. In addition, NADPH oxidase, a neuronal enzyme that produces ROS, is found in the hypothalamus, especially in the arcuate nucleus (ARC), ventromedial (VMN), and PVN regions (14, 17). The ARC, PVN, and VMN are known to contain neuromodulators that affect fertility (37). NPY/AgRP and POMC/CART neurons in the ARC project directly onto GnRH neuron cell bodies and nerve terminals (38, 39). Neuropeptides released by these neurons can influence GnRH neuron activity (40, 41). Furthermore, cellular activity of the NPY/AgRP and POMC/CART neuronal population is directly controlled by intracellular ROS (17). In the case of GnRH neurons, ROS H_2_O_2_ appeared to influence neuronal activity across postnatal development in a concentration-dependent and estrous-cycle-dependent manner.

Our findings, revealed that 1 mM H_2_O_2_ inhibited adult GnRH neurons, consistent with previous studies on dopamine neurons (23), PVN (18), substantia nigra pars reticulate (SNr) GABAergic neurons (35), and intrinsic cardiac ganglia neurons (42). Most studies using adult experimental animals have shown that H_2_O_2_ can inhibit neuronal excitability (18, 23, 35, 42). However, unlike adults, most immature GnRH neurons were stimulated by the same concentration of H_2_O_2_. According to previous studies, oxidative stress vulnerability increase with age, with young rats being more resistant to ROS than adults (30). Furthermore, H_2_O_2_ has both excitatory and inhibitory effects on neuronal excitability depending on neuronal population and brain location (43).

In the present study, the responsiveness of adult female GnRH neurons to H_2_O_2_ exposure varied throughout the estrous cycle. Circulating gonadal hormones, which fluctuate during estrous phases (44), can significantly impact GnRH neuronal excitability (45). Some studies show that proestrus mice had higher GnRH neuronal activity than mice in other estrous phases (46, 47). On the other hand, *Piet et al.* have reported less GnRH neuronal activity in proestrus mice than in mice at diestrus stage (48). According to previous studies, estradiol appears to have a positive feedback effect on GnRH neuronal activity in proestrus mice (49), and a neuroprotective effect against oxidative stress (50). We found that GnRH neurons in proestrus mice were more vulnerable to oxidative stress than those in estrous and diestrous stages. There is no information on how circulating steroid hormones influence GnRH neurons during oxidative stress. This requires further investigation.

In mature GnRH neurons, H_2_O_2_ mainly caused hyperpolarization and action potential suppression. Such H_2_O_2_-mediated response was retained in the presence of voltage-gated Na^+^ channel blocker TTX and AARBC, indicating a post-synaptic effect of H_2_O_2_ on GnRH neurons. H_2_O_2_ has been previously shown to have a similar post-synaptic effect (18). Studies have shown that H_2_O_2_ can induce membrane potential depolarization and hyperpolarization *via* different mechanisms. H_2_O_2_ can activate transient receptor potential channels (35, 51) or inhibit inward-rectifying K^+^ channels to induce depolarization (52). Opening of K_ATP_ channels leads to hyperpolarization (18, 23, 35). Activation of barium-sensitive potassium channels by H_2_O_2_ exposure has also been reported in a few studies (53). Similar to other studies, we observed the involvement of K_ATP_ and Ba^2+^ sensitive potassium channel in the hyperpolarization of GnRH neurons induced by H_2_O_2_.

The potassium channel plays a role in hormone and neurotransmitter release (54). Identifying signaling molecules that affect K^+^ channels in GnRH neurons is of particular interest nowadays. Studies have shown that GnRH neurons are susceptible to metabolic stress, which activates K_ATP_ channels. Functional K_ATP_ channel subunits have been detected in GnRH neurons (55). When the ATP/ADP ratio falls, K_ATP_ channels, which govern resting membrane properties of neurons, will open, causing cells to hyperpolarize and provide neuroprotection (56). Aside from neuroprotection, K_ATP_ channels are involved in glucose homeostasis in the hypothalamus, including GnRH neurons (55, 57). Recently, H_2_O_2_ has been identified as a signaling molecule for K_ATP_ channel activation (23, 35). Furthermore, inhibiting GPx and CAT of antioxidant systems can increase endogenous H_2_O_2_ in midbrain dopamine neurons (23) and SNr GABAergic neurons (35), resulting in K_ATP_ channel activation.

GPx and CAT are two major enzymes involved in H_2_O_2_ detoxification. Therefore, antioxidant enzymes inhibitors ATZ and MCS were used to determine the effect of endogenous H_2_O_2_ on GnRH neuronal excitability in the present study. ATZ is a CAT inhibitor that elevates endogenous H_2_O_2_ (58). It has a similar effect as exogenous H_2_O_2_ on midbrain dopamine neurons (23). However, ATZ showed no effect on GnRH neuron excitability. On the other hand, inhibition of GPx, another antioxidant enzyme, caused GnRH neurons to lose their spontaneous activity. *Avshalumov et al.* have reported a similar result. They showed that MCS treatment caused most dopamine neurons in the midbrain to hyperpolarize and lose their spontaneous activity (23). CAT and GPx are endogenous antioxidant-active enzymes responsible for the enzymatic clearance of H_2_O_2_, changing H_2_O_2_ into H_2_O and O_2_ molecules (18, 59). GPx is a crucial enzyme in the cytosol that plays an important role in the host’s defense against oxidative stress (60). Its principal antioxidant enzyme activity is to protect neurons against H_2_O_2_ toxicity (61). CAT is predominantly found in peroxisomes while GPx is distributed in the cytosol and mitochondria (61). Inhibiting GPx may cause H_2_O_2_ to accumulate in the cytosol, hence regulating neuronal excitability.

GnRH neurons not only can respond to hormonal, neurotransmitter, and neuropeptide inputs, but also can react directly to metabolic signals (55, 62, 63). The generation of reactive oxygen species is commonly linked to metabolic signals. In aging and pathologic situations, impairment in the antioxidant defense system becomes more noticeable, resulting in increased ROS generation (64, 65). The interaction between energy metabolism and ROS becomes more evident during aging, increasing the risk of age-related illnesses (66). Female reproductive disorders such as endometriosis, polycystic ovary syndrome, preeclampsia, and recurrent pregnancy loss can result from a pro-oxidant/antioxidant imbalance (12). Similarly, oxidative stress can affect sperm function in males, resulting in infertility (67). We demonstrated that H_2_O_2_ inhibited the majority of adult GnRH from both sex, which could reinforce the preexisting hypothesis about oxidative stress is linked to infertility. Furthermore, the direct impact of H_2_O_2_ on GnRH neuronal excitability *via* ion-channel mechanism could explain the cause of ROS disruption in the crosstalk of the HPG axis with another endocrine axis at hypothalamic levels and ROS-induced hormonal imbalance that leads to infertility.

In conclusion, current findings indicate that H_2_O_2_ can regulate K_ATP_ channels in adult GnRH neurons. Potassium channels can influence hormone and neurotransmitter release. Thus, oxidative stress regulating K_ATP_ channels in hypothalamic GnRH neurons could modulate pulsatile release of gonadotropins, impacting the reproductive axis.

## Data availability statement

The raw data supporting the conclusions of this article will be made available by the authors, without undue reservation.

## Ethics statement

The animal study was reviewed and approved by Institutional Animal Care and Use Committee of Jeonbuk National University (CBNU 2020-0122).

## Author contributions

SR performed the experiments, analyzed the data, and wrote the draft. SJ contributed to reviewing and editing the draft. DC and SH conceptualized and design the study and completed the manuscript. All authors contributed to the article and approved the submitted version.

## Funding

This research was supported by the National Research Foundation of Korea (NRF) grant funded by the Korean government (MSIT) (2021R1F1A1045406) and (2021R1F1A1046123). The funders had no role in the design, analysis, or writing of this article.

## Conflict of interest

The authors declare that the research was conducted in the absence of any commercial or financial relationships that could be construed as a potential conflict of interest.

## Publisher’s note

All claims expressed in this article are solely those of the authors and do not necessarily represent those of their affiliated organizations, or those of the publisher, the editors and the reviewers. Any product that may be evaluated in this article, or claim that may be made by its manufacturer, is not guaranteed or endorsed by the publisher.
